# Lignocellulosic xylitol production from corncob using engineered *Kluyveromyces* *marxianus*

**DOI:** 10.3389/fbioe.2022.1029203

**Published:** 2022-10-21

**Authors:** Jia Zhang, Teng Xu, Xiaohang Wang, Xiaoyan Jing, Jia Zhang, Jiong Hong, Jian Xu, Jichao Wang

**Affiliations:** ^1^ Single-Cell Center, CAS Key Laboratory of Biofuels and Shandong Key Laboratory of Energy Genetics, Qingdao Institute of Bioenergy and Bioprocess Technology, Chinese Academy of Sciences, Qingdao, China; ^2^ Laboratory of Marine Biology and Biotechnology, Qingdao National Laboratory for Marine Science and Technology, Qingdao, China; ^3^ University of Chinese Academy of Sciences, Beijing, China; ^4^ State Key Laboratory of Microbial Technology, Shandong University, Qingdao, China; ^5^ School of Life Sciences, University of Science and Technology of China, Hefei, China; ^6^ Hefei National Laboratory for Physical Science at the Microscale, Hefei, China

**Keywords:** xylitol, xylose reductase, *Kluyveromyces marxianus*, corncob hydrolysate, crystallization

## Abstract

Xylitol production from lignocellulose hydrolysate is a sustainable and environment-friendly process. In this study, a systematic process of converting corncob waste into xylitol is described. First, the corncobs are hydrolyzed with acid to a hydrolysate. Second, *Kluyveromyces marxianus* YZJQ016 derived from *K. marxianus* YZJ074, constructed by overexpressing *ScGAL2-N376F* from *Saccharomyces cerevisiae*, *CtXYL1* from *Candida tropicalis*, and *KmZWF1* from *K. marxianus*, produces xylitol from the hydrolysate. A total of ten xylose reductase genes were evaluated, and *CtXYL1* proved best by showing the highest catalytic activity under the control of the *KmGAPDH* promoter. A 5 L fermenter at 42°C produced 105.22 g/L xylitol using *K. marxianus* YZJQ016—the highest production reported to date from corncob hydrolysate. Finally, for crystallization of the xylitol, the best conditions were 50% (v/v) methanol as an antisolvent, at 25°C, with purity and yield of 99%–100% and 74%, respectively—the highest yield reported to date.

## Introduction

The five-carbon sugar alcohol, xylitol, has attracted great attention recently. It is one of the top 12 value-added compounds from biomass identified by the U.S. Department of Energy (Zhang et al., 2011) with a wide variety of food, pharmaceutical, and odontological applications because of sweetening similar to sucrose, but with fewer calories (Oh et al., 2007; Ling et al., 2011).

Currently, xylitol is mainly produced by hydrogenation of d-xylose using nickel catalysts, with drawbacks in energy consumption, wastewater pollution, and extensive purification requirements (Atzmuller et al., 2020). Highly efficient biotechnological approaches, using microorganisms, are being developed as alternative processes (Su et al., 2013). Microorganisms can convert lignocellulose biomass into bioenergy and chemicals by utilizing glucose and xylose. Yeasts are considered to be the most efficient producers of xylitol among these microorganisms (Guo et al., 2006). *K. marxianus* is a “generally regarded as safe” (GRAS) yeast, which has the fastest-growing rate of eukaryotic organisms (Zhou et al., 2018). Due to the thermotolerance of *K. marxianus*, cooling costs and the risk of contamination can be reduced, so *K. marxianus* has attracted increased attention (Zhang et al., 2016).

Corncobs are abundant agricultural wastes, and more than 500 million tons are produced in China every year (Wang et al., 2013). Corncobs contain approximately 35%–40% hemicellulose and can be easily hydrolyzed to constituent carbohydrates, such as xylose (Ling et al., 2011; Ramesh et al., 2013). Thus, corncobs are an ideal and promising raw material to manufacture xylitol by bio-conversion (Ling et al., 2011; Ramesh et al., 2013).

For biotechnological xylitol production from corncobs, there are three key steps: corncobs hydrolysate, xylose fermentation, and xylitol purification (Hernandez-Perez et al., 2019). Although there are reports of useful enzymatic hydrolysis methods, they are not suitable for industrial production due to their high cost, time consumption, low carbohydrate content, and low efficiency (Guo et al., 2018). Dilute acid hydrolysis for xylitol production is a low-cost, fast, highly effective, and easily operable method to remove hemicellulose (Kumar and Sharma, 2017), although waste disposal needs to be investigated. High xylose conversion efficiency is important, and in recent years, genetic and metabolic engineering developments have increased production (Zhao et al., 2020). Due to low product concentrations and the complex composition of the fermentation broth, purification is a bottleneck that increases the costs and limits the yield of bioxylitol (Silva et al., 2020; Marques and Rocha, 2021). The subsequent purification of xylitol from fermented broth can comprise several steps, normally including concentration, clarification, and crystallization, so as to obtain high-purity xylitol at a commercial scale (Cardoso and Forte, 2021). The literature reports that xylitol crystallization has drawn more attention recently due to the simplicity of operation for high purity (Hartwig and Ulrich, 2016; Delgado et al., 2021). Most published studies described xylitol recovery yield between 40% and 60% and purity of less than 98% by crystallization from fermented hemicellulosic hydrolysates such as hardwood, corncobs, sugarcane bagasse, and synthetic mediums (Silva et al., 2020). However, purification must be performed before crystallization to obtain a highly purified and concentrated solution (Alves et al., 2021).

**FIGURE 1:**
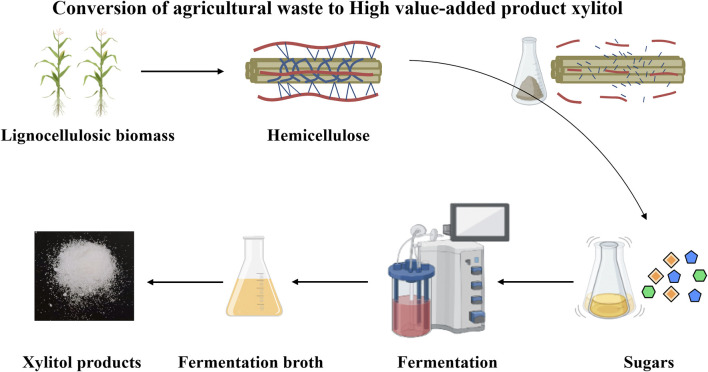
Systematic process from agricultural waste to high value-added product xylitol.

**FIGURE 2:**
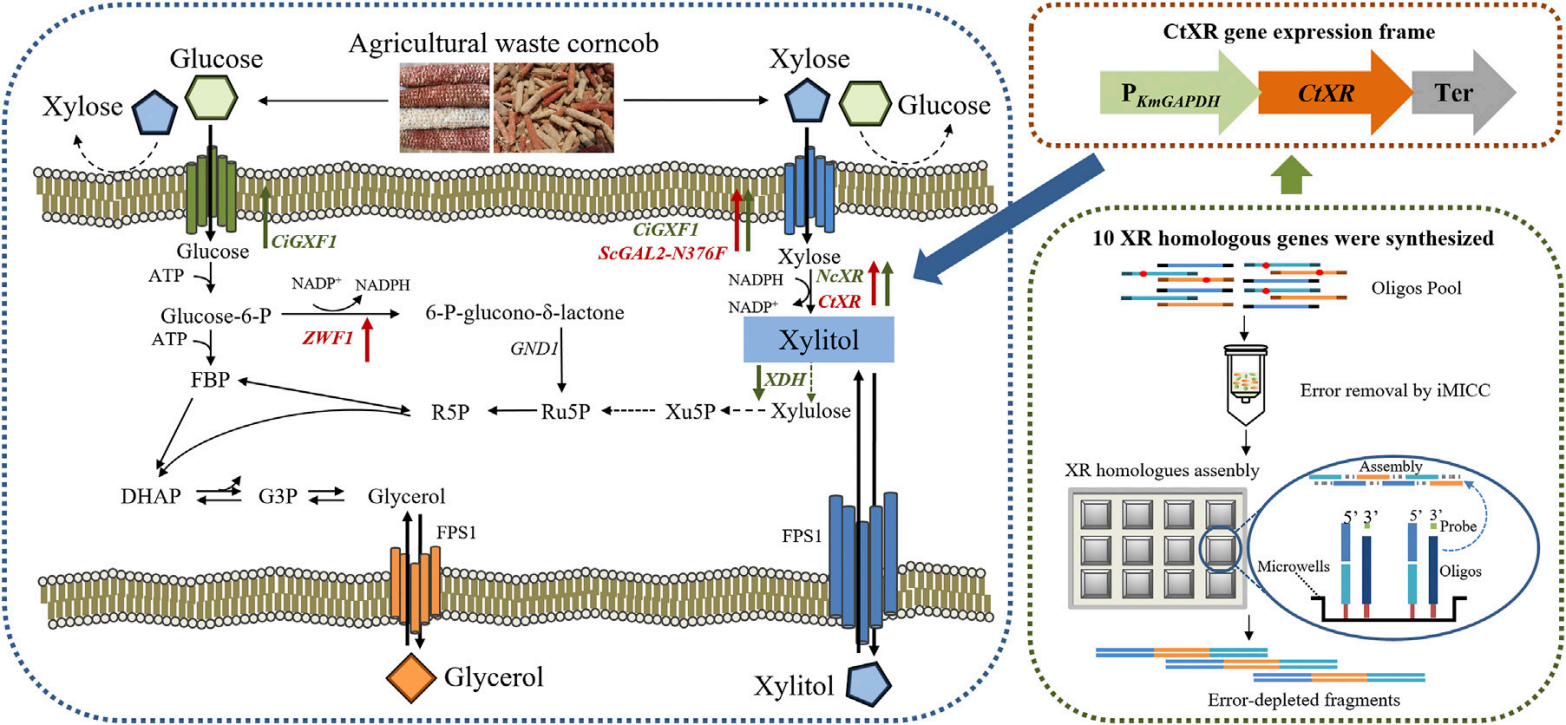
Schematic presentation of the metabolic pathway in modified *K. marxianus* strains. Upward arrows represent overexpression, and downward arrows represent disruption; the green ones were constructed in YZJ074 by Zhang et al. (2015), and the red ones were further constructed in this work.

To address the discussed challenges, we introduced and validated a systematic process for converting agricultural waste to xylitol (Figure 1). Corncobs were hydrolyzed into xylose and glucose. Several *K. marxianus* strains were constructed and tested for the fermentation of corncob hydrolysate, including the evaluation of ten XR (Xylose reductase) homologous genes (Zhang et al., 2020). YZJQ016, derived from *K. marxianus* YZJ074 (Figure 2), was used to produce xylitol in a 5 L fermentor at 42°C, and the highest xylitol production reported to date from corncob hydrolysate was obtained. Moreover, the highest purity and yield of xylitol from the fermentation broth were obtained by crystallization under optimized conditions. A platform for intelligent manufacturing of xylitol products by utilizing agricultural waste corncob *via* rewriting the metabolism pathway and optimizing the purification conditions was implemented.

## Materials and methods

### Reagents and microorganisms

All chemicals used were similar to those previously described (Zhang et al., 2016).

The yeast strains used in this study are listed in Table 1. *K. marxianus* YZB001 was a *Kmxyl1* disruption strain of *K. marxianus* YHJ010 (Zhang et al., 2011). *K. marxianus* YLUA005 was a *Kmxyl2* disruption strain of *K. marxianus* YHJ010 (Li et al., 2013); xylose cannot be consumed as xylose dehydrogenase (XDH) and was disrupted (Zhang et al., 2014). *K. marxianus* YZJ074 was constructed in our laboratory from *K. marxianus* YLUA005 (Zhang et al., 2014). Synthetic dropout (SD) medium was used to select the transformants, and yeast extract/peptone-dextrose (YPD) medium was used to culture *K. marxianus* strains aerobically, as previously described (Zhang et al., 2014). Fermentation and xylose assimilation ability was determined in YPXG medium (10 g/L yeast extract, 20 g/L bacterial peptone, and various amounts of d-xylose and d-glucose) and YPXG2 (10 g/L yeast extract, 20 g/L bacterial peptone, and various amounts of d-xylose, d-glucose, and glycerol). Agar (1.5%) was added to prepare solid plates of each medium. Gene cloning and expression vector construction were carried out in *Escherichia coli* XL10-Gold with Luria–Bertani (LB) media.

**TABLE 1:** Yeast strains used in this study.

Strain	Relevant genotype	Reference
YZB001	YHJ010, △*Kmxyl1*::*Sctrp1*	Zhang et al. (2011)
YLUA005	YHJ010, △*Kmxyl2*::*Sctrp1*	Li et al. (2013)
YZJ074	YHJ010, △*KmXYL2*::*ScTRP1*, 4 copies of *NcXYL1*, *CiGXF1*	Zhang et al. (2015)
YZJXR001	YZB001, pZJXR001, *Arxyl*, P_*ScGAPDH*_	This study
YZJXR002	YZB001, pZJXR002, *Abxyl*, P_*ScGAPDH*_	This study
YZJXR003	YZB001, pZJXR003, *Aoxyl*, P_*ScGAPDH*_	This study
YZJXR004	YZB001, pZJXR004, *Bbxyl*, P_*ScGAPDH*_	This study
YZJXR005	YZB001, pZJXR005, *Caxyl*, P_*ScGAPDH*_	This study
YZJXR006	YZB001, pZJXR006, *Cexyl*, P_*ScGAPDH*_	This study
YZJXR007	YZB001, pZJXR007, *Cgxyl*, P_*ScGAPDH*_	This study
YZJXR008	YZB001, pZJXR008, *Cpxyl*, P_*ScGAPDH*_	This study
YZJXR009	YZB001, pZJXR009, *Ctxyl*, P_*ScGAPDH*_	This study
YZJXR010	YZB001, pZJXR010, *Cmxyl*, P_*ScGAPDH*_	This study
YZJXR011	YLUA005, pZJXR009, *Ctxyl*, P_*ScGAPDH*_	This study
YZJXR012	YLUA005, pZJXR011, *Ctxyl*, P_*KmGAPDH*_	This study
YZJQ001	YZJ074, pZJ063, *ScGAL2*-N376F, *ScURA3, Zeocin* ^*R*^	This study
YZJQ002	YZJQ001, pZJ063, *ScGAL2*-N376F, *ScURA3, Zeocin* ^*R*^	This study
YZJQ003	YZJQ002, Δ*ScURA3*	This study
YZJQ004	YZJQ003, pZJ061, *ScGAL2*-N376F, *ScURA3*	This study
YZJQ005	YZJQ004, Δ*ScURA3*	This study
YZJQ010	YZJQ005, pZJXR011, *Ctxyl*, P_*KmGAPDH*_, *ScURA3, Zeocin* ^*R*^	This study
YZJQ011	YZJQ010, Δ*ScURA3*	This study
YZJQ016	YZJQ005, pZJ041, *KmZWF1*, *ScURA3, Zeocin* ^*R*^	This study

The cell concentration was determined by the absorbance at 600 nm using a NanoPhotometer NP80 spectrophotometer (Munich, Germany).

As previously described (Zhang et al., 2014), high-pressure liquid chromatography (HPLC) (Agilent 1100, United States) was used to determine the concentrations of d-xylose, xylitol, d-glucose, and glycerol with ROA-organic acid H^+^(8%) column (Phenomenex, United States); the mobile phase was 0.005 M H_2_SO_4_ with a flow rate of 0.3 ml/min. The column temperature was 75°C. To purify and crystallize xylitol from the fermentation broth of corncob hydrolysate, dichloromethane, methanol, and acetic acid were purchased from Fuyu Chemical (Tianjin, China). The rotary evaporator was obtained from Ruide (Henan, China). The rapid liquid phase preparation column SD-0000 and SL-8101 were purchased from Santai Technologies Inc. (Jiangsu, China). The xylitol crystal seeds were obtained from Sangon Biotech Co. (Shanghai, China).

### Construction of plasmids

The plasmids used in this study are listed in Table 2. The open reading frame (ORF) sequences of the ten XR homology genes (XRn) were designed and synthesized on-chip, and the error rates were reduced using our stability-improved iMICC system (Zhang et al., 2020). The resulting plasmids were named pMD18T-XRn and validated by sequencing (Zhang et al., 2020).

**TABLE 2:** Plasmids used in this study.

Plasmid	Selection marker and description	Reference
yEUGAP	*Scura3*, P_*ScGAPDH*_, T_*ScGAPDH*_	Zhang et al. (2015)
pMD18T-ΔScURA3	*Amp*, ΔScURA3	Zhang et al. (2015)
pZJ041	*Scura3*, P_*ScGAPDH*_-*KmZWF1*-T_*ScGAPDH*_	Zhang et al. (2016)
pZJ061	*Scura3*, P_*ScGAPDH*_-*ScGAL2-N376F*-T_*ScGAPDH*_	Zhang et al. (2016)
pZJ063	*ZeocinR,* P_*KmGAPDH*_-*ScGAL2-N376F*-T_*ScGAPDH*_	Zhang et al. (2016)
pMD18T-*Arxyl*	*Amp*, *Absidia repens*, *Arxyl*	Zhang et al. (2020)
pMD18T-*Abxyl*	*Amp*, *Acidobacteria bacterium*, *Abxyl*	Zhang et al. (2020)
pMD18T-*Aoxyl*	*Amp*, *Aspergillus oryzae*, *Aoxyl*	Zhang et al. (2020)
pMD18T-*Bbxyl*	*Amp*, *Brettanomyces bruxellensis*, *Bbxyl*	Zhang et al. (2020)
pMD18T-*Caxyl*	*Amp*, *Candida auris*, *Caxyl*	Zhang et al. (2020)
pMD18T-*Cexyl*	*Amp*, *Candida ergatensis*, *Cexyl*	Zhang et al. (2020)
pMD18T-*Cgxyl*	*Amp*, *Candida glycerinogenes*, *Cgxyl*	Zhang et al. (2020)
pMD18T-*Cpxyl*	*Amp*, *Candida parapsilosis*, *Cpxyl*	Zhang et al. (2020)
pMD18T-*Ctxyl*	*Amp*, *Candida tropicalis*, *Ctxyl*	Zhang et al. (2020)
pMD18T-*Cmxyl*	*Amp*, *Candida milleri*, *Cmxyl*	Zhang et al. (2020)
pZJXR001	*Scura3*, P_*ScGAPDH*_-*Arxyl*-T_*ScGAPDH*_	This study
pZJXR002	*Scura3*, P_*ScGAPDH*_-*Abxyl*-T_*ScGAPDH*_	This study
pZJXR003	*Scura3*, P_*ScGAPDH*_-*Aoxyl*-T_*ScGAPDH*_	This study
pZJXR004	*Scura3*, P_*ScGAPDH*_-*Bbxyl*-T_*ScGAPDH*_	This study
pZJXR005	*Scura3*, P_*ScGAPDH*_-*Caxyl*-T_*ScGAPDH*_	This study
pZJXR006	*Scura3*, P_*ScGAPDH*_-*Cexyl*-T_*ScGAPDH*_	This study
pZJXR007	*Scura3*, P_*ScGAPDH*_-*Cgxyl*-T_*ScGAPDH*_	This study
pZJXR008	*Scura3*, P_*ScGAPDH*_-*Cpxyl*-T_*ScGAPDH*_	This study
pZJXR009	*Scura3*, P_*ScGAPDH*_-*Ctxyl*-T_*ScGAPDH*_	This study
pZJXR010	*Scura3*, P_*ScGAPDH*_-*Cmxyl*-T_*ScGAPDH*_	This study
pZJXR011	*Scura3*, P_*KmGAPDH*_-*Ctxyl*-T_*ScGAPDH*_	This study

The primers used in this study can be found in Supplementary Table S1. The full-length DNA of XRn was then amplified with primers XRn-ECORI-F and XRn-NOTI-R and cloned to yEUGAP (gift from Hisanori Tamaki) between the *EcoR* I and *Not* I sites to obtain the plasmids pZJXR001 to pZJXR010, in which XR homology genes were expressed under the control of the *ScGAPDH* promoter. A fragment containing *Ctxyl1*-T_*ScGAPDH*_ was amplified from pZJXR009 with primers KMGAP-CTXR-F and TER-HINDIII-R. The promoter was amplified from pMD18T-P_*KmGAPDH*_ (Zhang et al., 2014) and fused with the *Ctxyl1*-T_*ScGAPDH*_ fragment through OE-PCR with primers KMGAP-HINDIII-F and TER-HINDIII-R. The fused fragment, which contained the promoter P_*KmGAPDH*_ and *Ctxyl1*-T_*ScGAPDH*_, was inserted into yEUGAP, which contained *Scura3* at the *Hind* III site.

In brief, pZJXR001 was constructed for the expression of *Absidia repens* xylose reductase gene (*Arxyl*); pZJXR002 was constructed for the expression of *Acidobacteria bacterium* xylose reductase gene (*Abxyl*); pZJXR003 was constructed for the expression of *Aspergillus oryzae* xylose reductase gene (*Aoxyl*); pZJXR004 was constructed for the expression of *Brettanomyces bruxellensis AWRI* xylose reductase gene (*Bbxyl*); pZJXR005 was constructed for the expression of *Candida auris* xylose reductase gene (*Caxyl*); pZJXR006 was constructed for the expression of *Candida ergatensis* xylose reductase gene (*Cexyl*); pZJXR007 was constructed for the expression of *Candida glycerinogenes* xylose reductase gene (*Cgxyl*); pZJXR008 was constructed for the expression of *Candida parapsilosis* xylose reductase gene (*Cpxyl*); pZJXR009 was constructed for the expression of *C. tropicalis* xylose reductase gene (*Ctxyl*); pZJXR010 was constructed for the expression of *Candida millerii* xylose reductase gene (*Cmxyl*); and pZJXR011 was constructed for the expression of *C. tropicalis* xylose reductase gene (*Ctxyl*) under the control of the *KmGAPDH* promoter. A schematic diagram of the plasmids and strains construction is shown in Supplementary Figure S1.

### Construction of xylitol-producing strains

The plasmids were linearized and transformed into *K. marxianus* YZB001 or YLUA005 (Table 1), as previously described (Abdel-Banat et al., 2010; Zhang et al., 2013). These strains harbored different *XYL1* genes and were used to evaluate their function in the improvement of corncob hydrolysate fermentation. The transformants of all strains except pZJ063 were selected on SD medium without uracil for 2 days at 37°C. The transformants of pZJ063 were selected on SD medium with zeocin for 2 or 3 days at 37°C. All integrations of XR genes were confirmed by PCR (Zhang et al., 2013). To express heterogeneous genes in YZJQ002, YZJQ004, or YZJQ010, a ScURA3 disruption cassette was amplified from pMD18T-ΔScURA3 (Farwick et al., 2014; Zhang et al., 2015) and transformed to disrupt ScURA3 in YZJQ002, YZJQ004, or YZJQ010 (Zhang et al., 2014). The URA3 disrupted strain was selected on an SD plate containing uracil and 0.1% 50-fluoro-orotic acid (5′-FOA) and named YZJQ003, YZJQ005, or YZJQ011. The obtained strains, which were transformed with different XR genes, were named as described in Table 1.

### Comparison of different XRs with different promoters for xylitol production

To compare the function of different xylose reductases and the promoters P_*KmGAPDH*_ and P_*ScGAPDH*_ in xylitol production, strains YZJXR001, YZJXR002, YZJXR003, YZJXR004, YZJXR005, YZJXR006, YZJXR007, YZJXR008, YZJXR009, and YZJXR010, which were derived from YZB001 (Table 1), were fermented at 42°C with YP medium (10 g/L yeast extract and 20 g/L bacterial peptone) containing 50 g/L xylose. YZJXR011 and YZJXR012, which were derived from YLUA005 (Table 1), were fermented at 42°C with YP medium containing 50 g/L xylose and 20 g/L glycerol.

Unless otherwise indicated, all fermentations were performed in a 250-ml Erlenmeyer flask containing 30 ml media shaken at 250 rpm with initial OD_600_ = 1 pre-cultured cells, and all results were performed in triplicate and reported as the mean values.

### Comparison of the fermentation of the strains with the various *ScGAL2-N376F* copies

YZJQ001, YZJQ002, and YZJQ004 constructed from *K. marxianus* YZJ074, which contain different copies of *ScGAL2-N376F*, were fermented at 42°C with YP media containing about 80 g/L xylose and 20 g/L glucose to compare the effects of *ScGAL2-N376F* copy number on the fermentation.

### Evaluation of the glucose–xylose co-fermentation ability with further engineered *K. marxianus* strains

*K. marxianus* YZJQ004 and its further engineered strains, including YZJQ010 and YZJQ016, were fermented with the YP medium containing 100 g/L xylose, 10 g/L glucose, and 20 g/L glycerol.

### Preparation of corncob hemicellulose hydrolysate

The corncob hydrolysate was prepared as previously described (Cheng et al., 2014; Zhang et al., 2016). Acid hydrolysis of corncobs was performed at 127°C with dilute acid (0.5% (w/w) H_2_SO_4_ + 1.5% (w/w) H_3_PO_4_) for 1 h using a solid: liquid (quality: volume) ratio of 1:4. The hydrolysate was over-limed with Ca(OH)_2_ to prepare the detoxified material as previously reported with some modifications (Martinez et al., 2000; Verbeke et al., 2011). In brief, after acid pretreatment, the hydrolysate was separated with filtration, heated to 60°C, and Ca (OH)_2_ was added and mixed for 30 min, and the pH was adjusted to 6.0 and centrifuged to remove the precipitation. Activated carbon was added to the supernatant at 37°C for 24 h to adsorb color and was then subjected to vacuum filtration through 0.45 μm filters to separate the corncob hydrolysate. The over-limed hydrolysate solution was concentrated and mixed with various ratios for fermentation.

### *K. marxianus* YZJQ016 fermented high-concentration corncob hydrolysate using a fermenter

*K* *. marxianus* YZJQ016 were cultivated in YP media containing detoxified corncob hydrolysate with final concentrations of about 73.46 g/L xylose and 7.11 g/L glucose, and 20 g/L glycerol was added. YZJQ016 was pre-cultivated overnight at 37°C and inoculated into the fermentation medium with an initial OD600 of 1. The fermentations were performed at 42°C using a 5 L fermentor (Baoxing Corp., Shanghai, China). The agitation and aeration rates were maintained at 450 rpm and 1 vvm (volume of air per volume of medium per min), respectively (Zhang et al., 2014).

### Higher concentration of xylitol was obtained through fed-batch fermentation

To obtain a higher final xylitol concentration without sacrificing xylitol yield, fed-batch fermentations were performed. YZJQ016 was fermented with a high concentration of condensed detoxified corncob hydrolysate. The concentration of xylose and glucose in the treated corncob hydrolysate medium were 95.74 g/L and 9.95 g/L, and 31.53 g/L glycerol was added. At 30 h, 500 ml of detoxified corncob hydrolysate, non-sterilized yeast extract and glycerol were fed into the fermenter directly. Silicon defoamer (50%, v/v, prepared with distilled water) was added to control foaming.

### Xylitol purification

To purify and crystallize xylitol from the corncob hydrolysate, the fermentation broth was concentrated in a rotary evaporator. Furthermore, to promote xylitol crystallization, equal amounts of methanol were added to the concentrated solutions and shaken well in a constant-temperature water bath. The xylitol seed, which is conducive to seed nucleation, was added into the system at 4°C for 48 h to favor nucleation according to the ratio of concentrated solution to seed 200:1. After crystallization, the colored compound crystal and mother liquor (named mother liquor 1) were filtered from the crystallization solution through a sand core funnel. The rapid liquid phase preparation column SD-0000 and SL-8101 were used to purify the colored compounds crystal, and then the different components (dichloromethane, methanol, and acetic acid) of eluent in the ratio of (4:2:1) were used to separate the purified xylitol solution. The purified xylitol solution was concentrated, and the concentrated solution with xylitol crystal seeds was crystallized with a rotary evaporator (100 r/min) for 3 h and stood overnight at room temperature to obtain xylitol crystal (crystal 1) and crystallization mother liquor (named mother liquor 2). In order to obtain more xylitol crystals, the crystallization mother liquor 2 is concentrated and crystallized again to obtain xylitol crystal (crystal 2) and crystallization mother liquor (named mother liquor 3). The crystallization mother liquor 3 and the previously obtained crystallization mother liquor 1 were combined and concentrated to dry and then purified and crystallized again. The purification and crystallization method is as described previously to obtain xylitol crystal (named crystal 3) (Supplementary Figure S2). In the process of industrial production, the secondary purification can be purified together with the subsequent fermentation broth so as to reduce cost.

## Results and discussion

### Strains with different XRs were constructed, and the xylose-utilizing ability was evaluated

**FIGURE 3:**
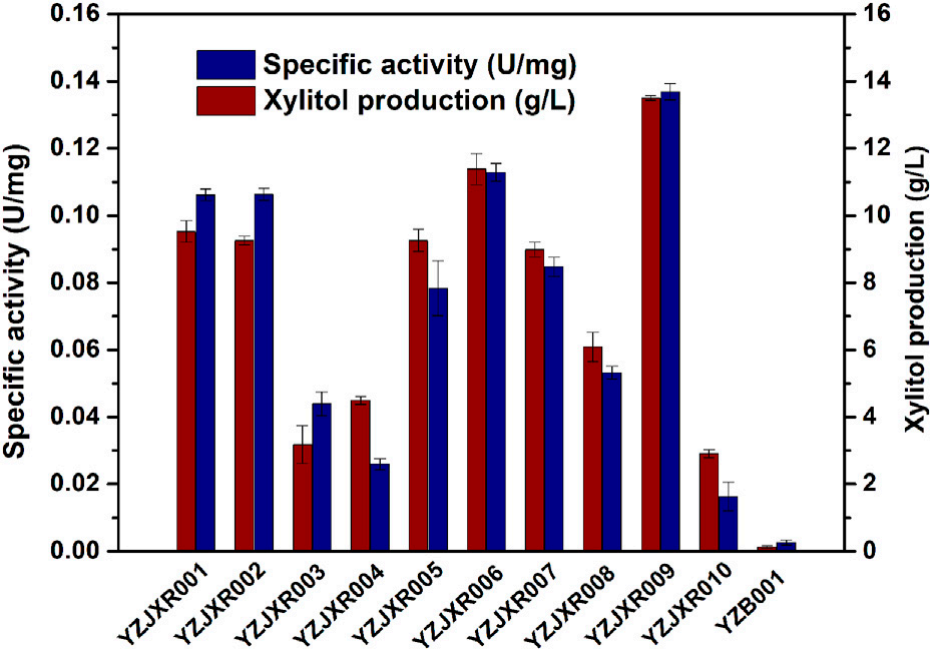
Comparison of XR activities of strains with different XRs.

Xylose reductase (XR) is a key component of the metabolic pathway that converts xylose to xylitol. Low expression and activity of xylose reductase in *K. marxianus* inhibits the accumulation of xylitol by fermentation (Rao et al., 2016; Dasgupta et al., 2017). In previous work, ten XR homologues were synthesized through the method of gene assembled on-chip and the stability-improved iMICC system (Zhang et al., 2020). To screen the one that could best further improve the xylose assimilation and xylitol production, the XR disrupted strain YZB001 was transformed with the pZJXR001, pZJXR002, pZJXR003, pZJXR004, pZJXR005, pZJXR006, pZJXR007, pZJXR008, pZJXR009, and pZJXR010 plasmids, respectively (Table 1). The strains expressing codon-optimization of the XR from *C. tropicalis* (*CtXR*) produced more xylitol than the other strains with 50 g/L xylose (Table 3), and the XR activity of each strain was determined to define the reason for the high xylitol production capacity of *CtXR* (Figure 3). Previous studies have shown that *CtXR* can be expressed in *E. coli* and improves xylose assimilation (Pal et al., 2016). In this study, *CtXR* demonstrated further improvement in xylose assimilation and xylitol production in *K. marxianus*.

**TABLE 3:** Fermentation of the strains harboring different XR genes under *Sc*GAPDH or *Km*GAPDH promoter.

Strain	Host strain	Promoter	XR	Xylitol production (g/L)	Xylitol productivity (g/L/h)
YZB001	_	_	_	0.13 ± 0.04	0.01 ± 0.01
YZJXR001	YZB001	P_*ScGAPDH*_	*Ar*XR	9.53 ± 0.02	0.40 ± 0.01
YZJXR002	YZB001	P_*ScGAPDH*_	*Ab*XR	9.26 ± 0.13	0.39 ± 0.03
YZJXR003	YZB001	P_*ScGAPDH*_	*Ao*XR	3.18 ± 0.07	0.13 ± 0.01
YZJXR004	YZB001	P_*ScGAPDH*_	*BbXR*	4.50 ± 0.12	0.19 ± 0.02
YZJXR005	YZB001	P_*ScGAPDH*_	*Ca*XR	9.26 ± 0.03	0.39 ± 0.03
YZJXR006	YZB001	P_*ScGAPDH*_	*Ce*XR	11.38 ± 0.47	0.47 ± 0.02
YZJXR007	YZB001	P_*ScGAPDH*_	*Cg*XR	8.99 ± 0.02	0.37 ± 0.02
YZJXR008	YZB001	P_*ScGAPDH*_	*Cp*XR	6.09 ± 0.43	0.25 ± 0.03
YZJXR009	YZB001	P_*ScGAPDH*_	*Ct*XR	13.50 ± 0.07	0.56 ± 0.01
YZJXR010	YZB001	P_*ScGAPDH*_	*Cm*XR	2.91 ± 0.02	0.12 ± 0.05
YLUA005*	_	_	_	15.20 ± 0.97	0.42 ± 0.02
YZJXR011*	YLUA005	P_*ScGAPDH*_	*Ct*XR	21.56 ± 0.17	0.60 ± 0.01
YZJXR012*	YLUA005	P_*KmGAPDH*_	*Ct*XR	25.04 ± 0.73	0.70 ± 0.02

*In the fermentation with these strains, 20 g/L glycerol was added in addition to 50 g/L xylose.

Moreover, more xylitol was produced by the strains with the *CtXR* expressed under the *KmGAPDH* promoter. YZJXR012, which harbored *CtXR* under the control of the *KmGAPDH* promoter, produced more xylitol (25.04 g/L xylitol from 50 g/L xylose with 0.70 g/L/h productivity) than *ScGAPDH* promoter (21.56 g/L xylitol with 0.60 g/L/h productivity) (Table 3).

### Improving the glucose–xylose co-fermentation ability in *K. marxianus* with the expression of multiple copies *ScGAL2-N376F* and *Km*GAPDH promoter-controlled *CtXR*

**FIGURE 4:**
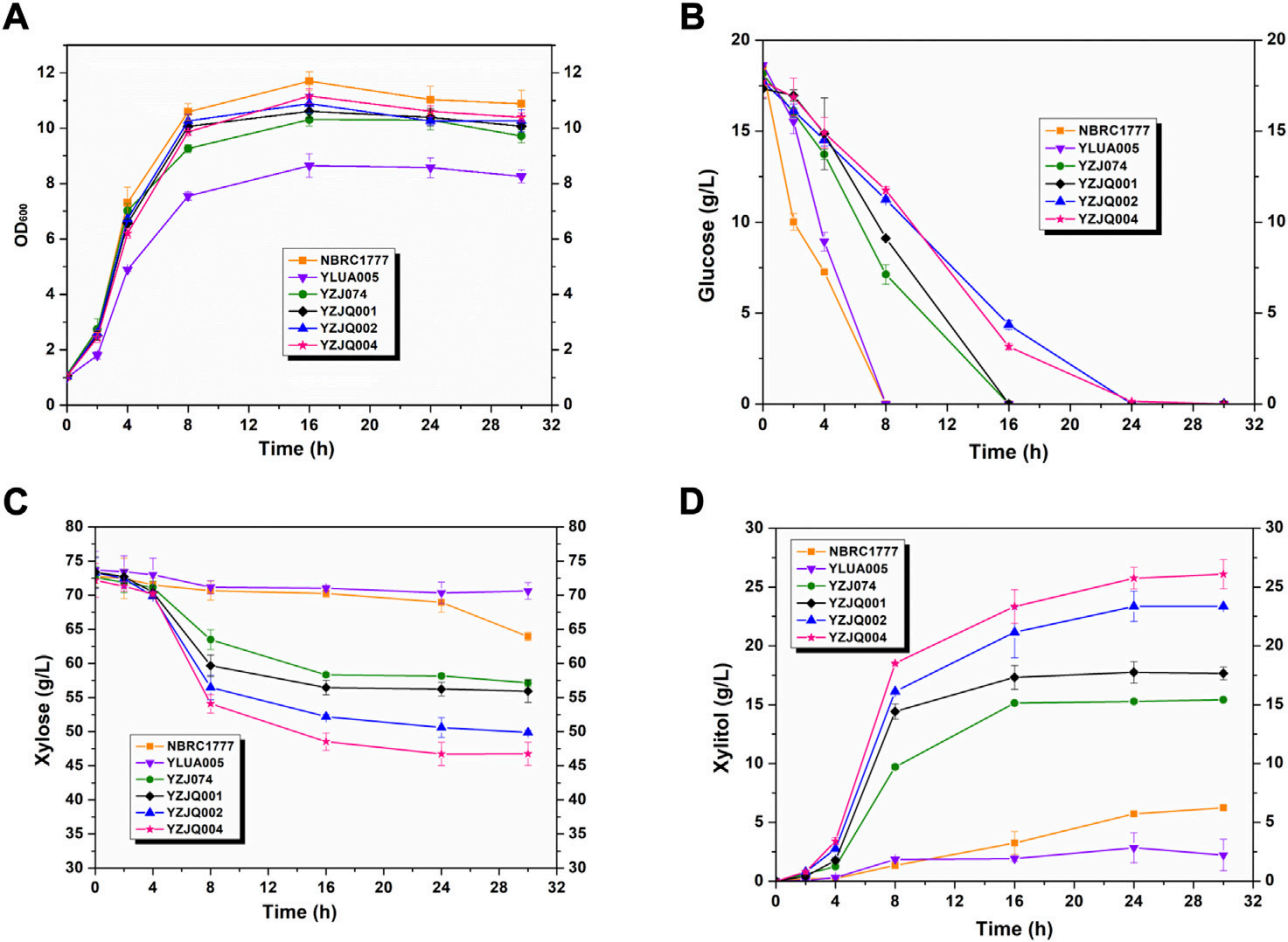
The glucose–xylose co-fermentation ability was improved by overexpression of ScGAL2-N376F. The OD600 **(A)**, glucose **(B)**, and xylose **(C)** consumption and xylitol **(D)** production of different strains with various genes fermented with YP medium containing about 80 g/L xylose and 20 g/L glucose at 42°C. The values are the means of three biological replicates ± standard deviation (*n* = 3) at each of the time points.

*K. marxianus* YZJ074 was constructed for xylitol production in our previous work (Zhang et al., 2015). YZJ074 utilized xylose to produce xylitol with glycerol as a co-substrate (Zhang et al., 2015). However, the glucose-repression effect on xylose limited the fermentation of corncob hydrolysate, which contains not only xylose but also glucose (Figure 4). The limiting factor is the specificity of xylose (Zhang et al., 2016). The N376F mutation of ScGAL2 (*ScGAL2*-N376F) was reported to be a d-xylose-specific transporter without d-glucose inhibition (Farwick et al., 2014; Zhang et al., 2016). To eliminate the glucose inhibitory effect, the xylitol production stage was expanded, and the fermentation efficiency of biomass was improved, for which various papers present methods, such as the overexpression of *ScGAL2N376F* and the deletion of *MIG1* (Berglund and Börjesson, 2006; Ghatak, 2011; Chandel et al., 2012; Wang et al., 2013; Zhang et al., 2016; Kumar and Sharma, 2017; Chandel et al., 2018; Hernandez-Perez et al., 2019; Arcano et al., 2020). In this study, YZJQ004, which expressed multi-copy of *ScGAL2-N376F*, produced xylitol in significantly greater amounts than other strains under the xylose and glucose co-fermentation, indicating that the *ScGAL2*-N376F mutant could transport xylose specifically with glucose present (Figure 4). When fermented with a 20 g/L glucose and 80 g/L xylose mixture, Figure 4A–C the xylitol productions in YZJQ001, YZJQ002, and YZJQ004 were 17.75, 23.36, and 25.75 g/L, respectively, when compared to YZ074 (15.28 g/L) (Figure 4D).

**FIGURE 5:**
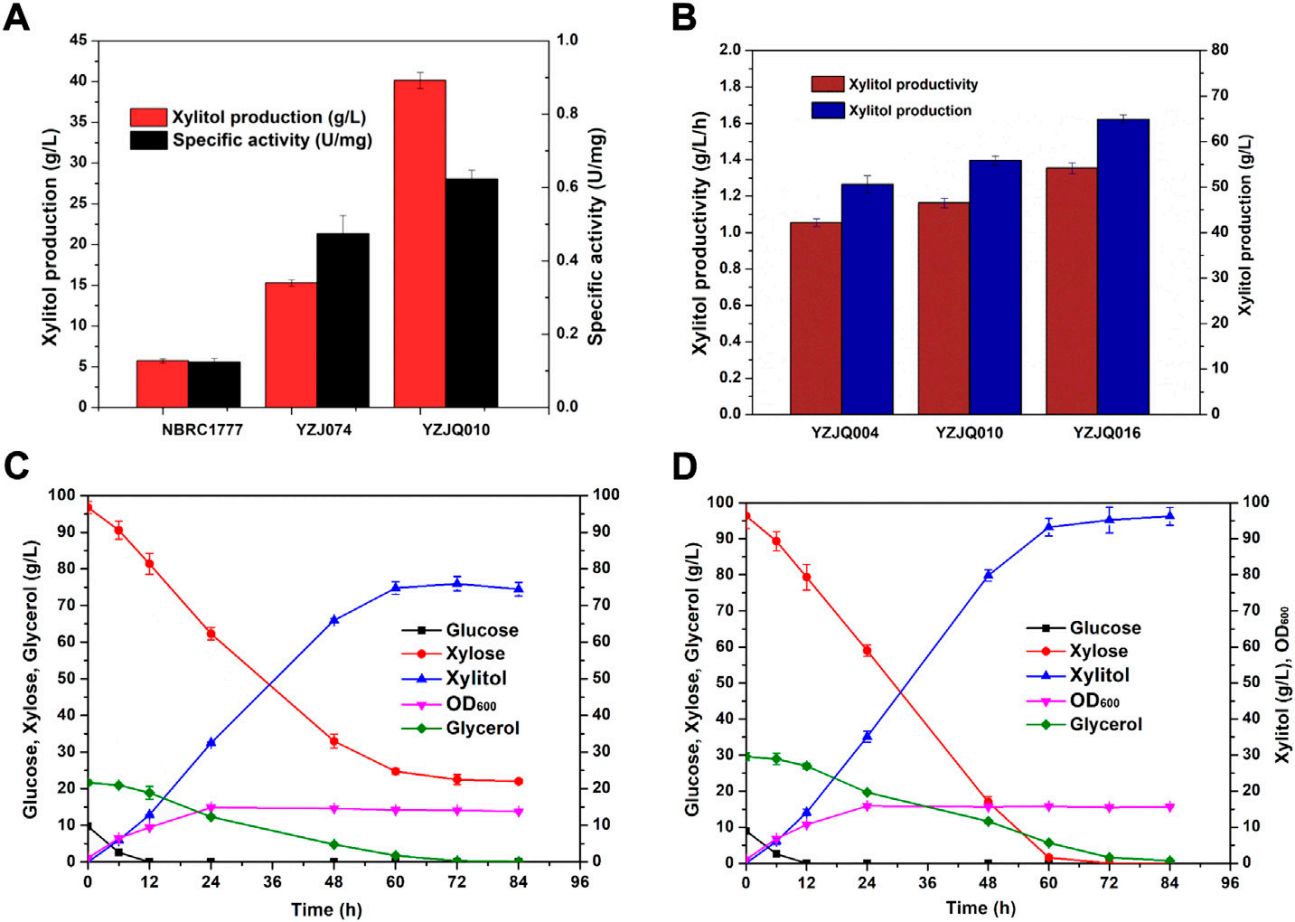
The glucose–xylose co-fermentation ability was further improved by keeping the redox balance. **(A)** The XR activity and xylitol production of different strains. **(B)** Co-fermentation of 10 g/L glucose, 100 g/L xylose, and 20 g/L glycerol at 42°C with YZJQ004, YZJQ010, or YZJQ016 for 48 h. **(C)** YZJQ016 fermented with 10 g/L glucose, 100 g/L xylose, and 20 g/L glycerol at 42°C. **(D)** YZJQ016 fermented with 10 g/L glucose, 100 g/L xylose, and 30 g/L glycerol at 42°C.

Additionally, YZJQ010 was obtained by overexpressing the *CtXR* gene controlled by the KmGAPDH promoter. YZJQ010 can significantly improve the production efficiency of xylitol at high xylose concentration, which indicates that the production efficiency of xylitol could be further improved by increasing the copies of exogenous XR genes (Figure 5B). To understand why overexpressing XR genes improves xylitol production, the XR activities were determined. As shown in Figure 5A, the XR activity of YZJQ010 (0.98/U mg) was higher than that of YZJ074 (0.38 U/mg). More copies of the XR gene could increase XR activity; therefore, more xylitol was produced with higher productivity.

### Improving the glucose–xylose co-fermentation ability by keeping the redox balance in YZJQ016 and evaluating its xylitol-producing ability with different ratios of sugars

In microbial processes, redox imbalance generated by NADPH-dependent XR is the major factor controlling xylitol accumulation (Yuan et al., 2019). Engineered *K. marxianus*, which utilized only NADPH as XR’s cofactor and were overexpressed copies of XR genes, has an imbalance cofactor metabolic system which limited the accumulation of xylitol. NADPH is largely consumed through overexpressing copies of *XYL1* genes, leading to the cofactors’ imbalance of the whole metabolic pathway. Cell growth depends on the necessary cofactors regenerated through different steps in the metabolic pathway. Therefore, the cofactors imbalance was considered the rate-limiting step in d-xylose fermentation and d-xylitol production (Salgado et al., 2012; Tani et al., 2017). The amount of xylose being converted to xylitol has to be well-balanced (Yang et al., 2020; Zhang et al., 2021). Researchers overexpressed the *S. cerevisiae* ZWF1 in the DWM strain or YJO-12 strain to increase the intracellular concentrations of NADPH so that the maximum yield of xylitol was increased (Oh et al., 2007; Jo et al., 2015). The overexpression of ZWF1 in *K. marxianus* YZJQ010 has also shown an effect in improving xylitol accumulation (Yuan et al., 2019). The engineered *K. marxianus* YZJQ010, named *K. marxianus* YZJQ016, enhanced both the production and productivity of xylitol by increasing intracellular concentrations of NADPH (Figure 5B). A possible explanation may be that the overexpression of *KmZWF1* led to keeping the redox balance in the cell.

To optimize the ratio of glucose, xylose, and glycerol for xylitol production, YZJQ016 was fermented with YP medium containing 10 g/L glucose, 100 g/L xylose, and 20- or 30-g/L glycerol. When 20 g/L glycerol was added into the medium, 74.76 g/L xylitol was produced, and 24.71 g/L xylose was unused in the medium at 60 h (Figure 5C). When 30 g/L glycerol was added, 96.21 g/L xylitol was produced from 96.35 g/L xylose with a yield on xylose of 0.99 g/g (Figure 5D). These results show that at least 30 g/L glycerol was needed for the reduction of 100 g/L xylose; the xylose/glucose/glycerol ratio was 10:1:3. This ratio was used for further research after comprehensive consideration.

### High-concentration xylitol produced from condensed corncob hydrolysate by YZJQ016 in a fermenter

**FIGURE 6:**
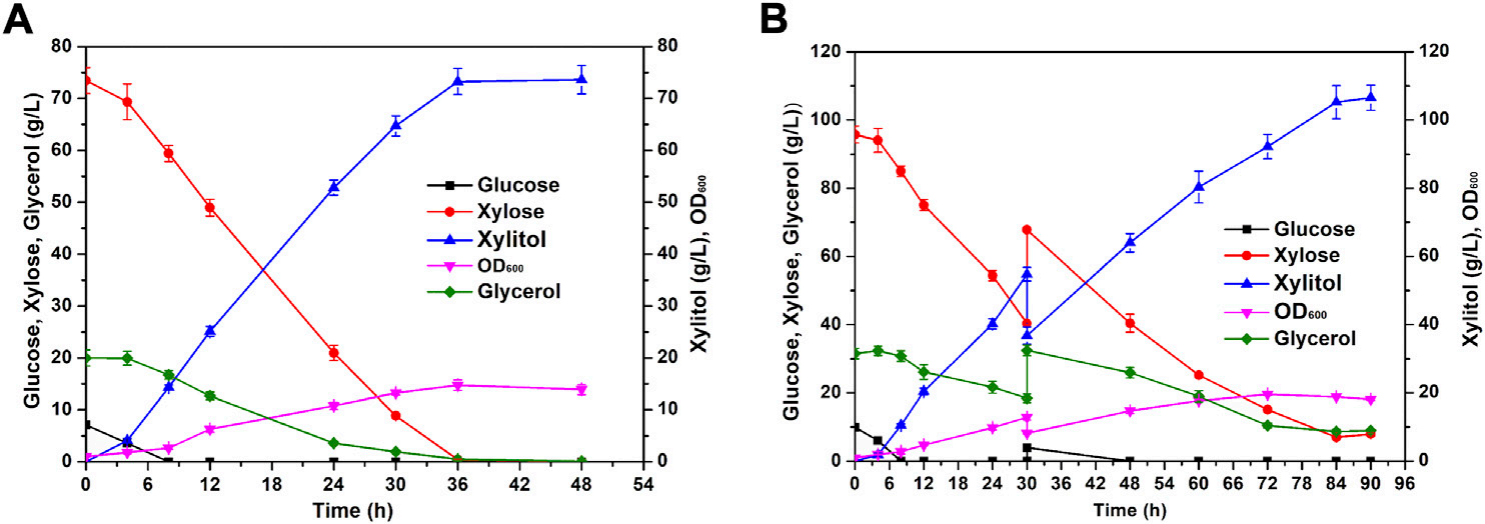
High-concentration xylitol produced from condensed corncob hydrolysate. **(A)** YZJQ016 fermented with concentrated corncob hydrolysate and 20 g/L glycerol in a fermenter at 42°C. **(B)** YZJQ016 fermented with concentrated corncob hydrolysate and 31.53 g/L glycerol for fed-batch at 42°C.

To integrate the utilization of corncobs, the diluted acid pretreated corncob hydrolysate was used to evaluate the co-fermentation ability of YZJQ016 in a fermenter. To evaluate the tolerance of YZJQ016 to the high concentration of detoxified corncob hydrolysate, YZJQ016 was fermented with YP medium containing condensed corncob hydrolysate, with glucose and xylose up to about 7.11 g/L and 73.46 g/L, respectively. As shown in Figure 6, YZJQ016 produced 73.27 g/L xylitol with a productivity of 2.04 g/L/h in 36 h when 73.05 g/L xylose was used (Figure 6A). Moreover, YZJQ016 produced 105.22 g/L xylitol with a productivity of 1.25 g/L/h in fed-batch fermentation (Figure 6B).

Up to now, the highest production and productivity of xylitol reported were 96.5 g/L and 1.2 g/L/h (0.45–0.93 g/g) when using batch fermentation in fermenters around 30°C (Table 4). The 105.22 g/L, 2.04 g/L/h, and 0.99 g/g in this study are all the highest xylitol production, productivity, and yield reported to date from the corncob hydrolysate (Table 4), in addition to the high temperature of 42°C with advantages as described in the introduction.

**TABLE 4:** Comparison of xylitol production of some yeast strains with corncob hydrolysate.

Strain	Fermentation time (h)	Temperature (°C)	Fermentation substrate	Xylitol yield (g/g)	Xylitol production (g/L)	Xylitol productivity(g/L/h)	References
*C. tropicalis* MTCC 6192^b^	24	30	Corncob	0.66	33.4	1.2	Kumar et al. (2018)
*K. marxianus* CICC 1727–5^a^	120	40	Corncob	0.82	24.2	NR	Du et al. (2020)
*S. cerevisiae* XP-RTK^a^	144	30	Corncob	NR	47	0.319	Kogje and Ghosalkar (2017)
*C. tropicalis* As 2.1776^a^	96	30	Corncob	0.74	58.3	0.61	Li et al. (2012)
	120			0.83	96.5	1.01	
*S. cerevisiae* YRH 396^b^	96	28	Corncob	0.45	8.17	NR	Cortivo et al. (2018)
*S.cerevisiae* PE-2^a^	96	30	Corncob	0.93	29.6	0.54	Baptista et al. (2018)
YZJQ016^b^	36	42	Corncob	0.99	73.05	2.04	This study
YZJQ016^a^	84	42	Corncob	0.94	105.22	1.25	This study

^a^ Fed-batch fermentation.

^b^ Fermentation directly.

So far, there are many studies on the conversion of agricultural waste into valuable product xylitol. Du et al. (2020) have developed a two-stage fermentation strategy to remove the inhibition of glucose on xylose and produce 24.2 g/L xylitol in a 5 L fermenter at 40°C by *K. marxianus* CICC 1727-5. Li et al. (2012) achieved high xylitol productivity in a two-stage fed-batch fermentation process by *C. tropicalis*, producing a maximum of 96.5 g/L of xylitol with a yield of 0.83 g/g in a 3.7 L fermentor. Kogje and Ghosalkar (2017) attained 47 g/L of xylitol by using glycerol as a co-substrate with a recombinant *S. cerevisiae* XP-RTK overexpressing the GRE3 endogenous gene. In our study, the xylose reductase genes were controlled under the *KmGAPDH* promoter and *KmZWF1* was overexpressed in the YZJQ016 and produced 105.22 g/L xylitol in a 5 L fermenter at 42°C.

### Xylitol purification from condensed corncob hydrolysate fermented by YZJQ016

**FIGURE 7:**
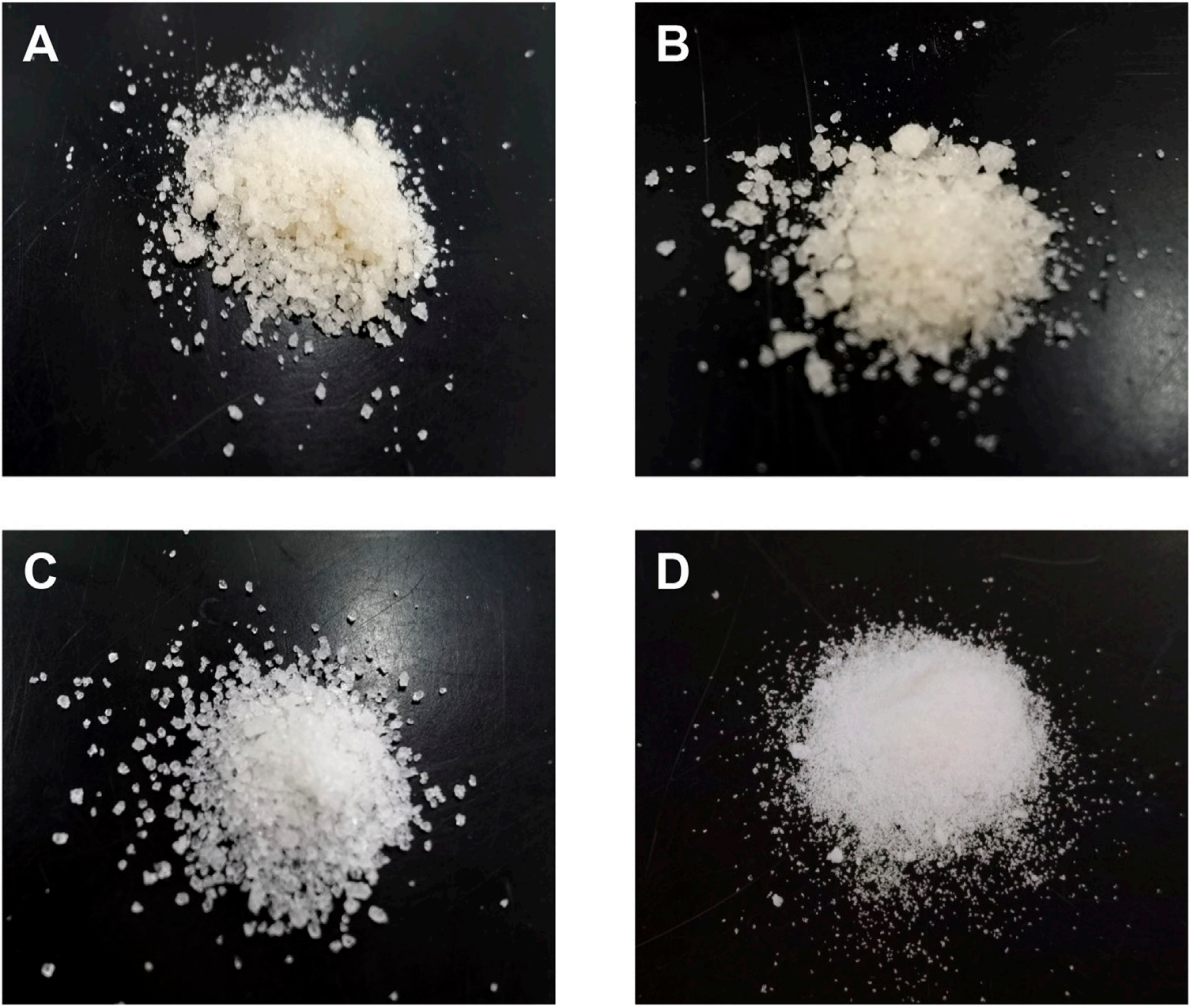
Images of xylitol crystals obtained. **(A)** Xylitol obtained by crystallization of the fermentation broth using 50% (v/v) methanol with a crystallization temperature of 0°C; **(B)** xylitol obtained by crystallization of the fermentation broth using 25% (v/v) methanol with a crystallization temperature of 25°C; **(C)** xylitol obtained by crystallization of the fermentation broth using 50% (v/v) methanol with a crystallization temperature of 25°C; **(D)** xylitol obtained by further secondary crystallization.

An economical and environment-friendly method for xylitol purification and crystallization from corncob hydrolysate was reported (Jinchao et al., 2010). However, the purity and yield of the crystalline xylitol obtained were only 95% and 60.2%, respectively. Studies have shown that the use of anionic and cationic resins to purify xylitol results in a yield loss of about 40%–55% due to the attachment of xylitol to the resin surface (Gurgel et al., 1995). Marques and Rocha (2021) used 50% (v/v) isopropanol as an antisolvent to reduce the loss of xylitol and yield 69.7%, but the purity was only 84.8%. Research studies have described crystallization methods for recovery of xylitol from fermentation broth (hardwood, corncob, sugarcane bagasse, and hemicellulose hydrolysates from synthetic media) with yields ranging from 40% to 60% and purity of less than 98% (Martínez et al., 2015). We used methanol as an antisolvent and iterative purification method to crystalize xylitol to improve the yield of xylitol. Appropriate methanol concentration and crystallization temperature improve the purity of xylitol (Martinez et al., 2008). Specifically, different concentrations (v/v) of methanol (25% and 50%) were used as the antisolvent, and different crystallization temperatures (0°C and 25°C) were set. Both the low concentration of methanol (25%) and low crystallization temperature (0°C) may cause the crystal color to turn yellow because the pigment will be entrained during crystallization (Figure 7A,B). The high concentration of methanol and high crystallization temperature both increased the solubility of xylitol and reduced the yield of xylitol. Based on these results, the crystallization process using 50% (v/v) methanol with a crystallization temperature of 25°C resulted in a purity of 99% (Figure 7C). Additionally, secondary crystallization will further improve the purity of xylitol (∼100%), which is the highest purity reported to date (Figure 7D). In contrast with other purification methods of xylitol produced by fermentation (Hong et al., 2007; Zhang et al., 2011; Li et al., 2013), this process has the advantage of being performed in an iterative purification method and having a higher yield (74%), which is the highest yield reported to date.

Marques and Rocha found that while using 50% (v/v) isopropanol as an antisolvent, the cooling rate was 0.5°C/min, and the yield and purity of xylitol secondary nucleation from hemicellulose hydrolysate were 69.7% and 84.8%, respectively (Marques and Rocha, 2021). Martinez et al. studied the combined effects of saturation temperature (40, 50, and 60°C) and cooling rate (0.10, 0.25, and 0.50°C/min). The results show that in ethanol/water (50:50% w/w) solution, when the cooling rate increases from 0.10 to 0.50°C/min, crystallization occurs very quickly (900–16,440 s) (Martinez et al., 2008). Vyglazov studied that the increase of ethanol concentration from 60% to 90% (v/v) accelerated the crystallization of xylitol at 25°C, while a simultaneous increase in temperature from 5 to 40°C led to a 7–10 fold increase in the rate of xylitol crystallization (Vyglazov, 2004). Crystallization using methanol as the counter solvent is more economical than other crystallization methods because our method only requires room temperature and 4°C, and does not require more energy to maintain high temperature and control the cooling rate.

To achieve the biological production of xylitol, there are still some challenges to overcome. Recovery and purification of xylitol is a complicated step, which is determined by the nature of complex components in the fermentation broth. Current methods can only extract part of xylitol (<74%), so better procedures or methods are needed to improve the purification and crystallization efficiency of xylitol (Rao et al., 2016; Hernandez-Perez et al., 2019).

## Conclusion

In this study, *K. marxianus* strains were constructed through metabolic engineering for high-efficiency xylitol production from corncob hydrolysate. Strain YZJQ016 produced 73.05 g/L xylitol from corncob hydrolysate, containing 73.46 g/L xylose, at 42°C. The 105.22 g/L, 2.04 g/L/h, and 0.99 g/g in this study are all the highest xylitol production, productivity, and yield from corncob hydrolysate reported to date, in addition to the high temperature of 42°C. Notably, the best purification conditions were 50% (v/v) methanol as an antisolvent and 25°C crystallization temperature, with the purity and yield of 99%–100% and 74%, respectively, which is the highest yield reported to date.

## Data Availability

The original contributions presented in the study are included in the article/Supplementary Material; further inquiries can be directed to the corresponding author.

## Supplementary material

Supplementary Table S1 Primers used in this study.

Supplementary Figure S1 Plasmids schema used in this study.

Supplementary Figure S2 Xylitol purification flow chart in this study.
