# Supplementary material for: Lignocellulosic xylitol production from corncob using engineered Kluyveromyces marxianus
Source: Front Bioeng Biotechnol. 2022 Oct 21;10:1029203. doi: 10.3389/fbioe.2022.1029203 (PMC9633946; doi:10.3389/fbioe.2022.1029203)
Supplement: Supplementary file 1 [file DataSheet1.docx]

Supplementary Material

**Table S1.** **The primers used in this study.**

| **Primer names** | **Primer sequences** |
| --- | --- |
| XR1-ECORI-F | CCGGAATTCATGACCATCGAATACCTGCA |
| XR1-NOTI-R | AAGGAAAAAAGCGGCCGCttaGTAGAAGATCGGCAGTTTCT |
| XR2-ECORI-F | CCGGAATTCATGCCGATCGCCCCTCCGTC |
| XR2-NOTI-R | AAGGAAAAAAGCGGCCGCttaACCTTCGATAGATTCGATCA |
| XR3-ECORI-F | CCGGAATTCATGGCGTCTCCGACCGTTAA |
| XR3-NOTI-R | AAGGAAAAAAGCGGCCGCttaGAAGATCGGAACGTACATAC |
| XR4-ECORI-F | CCGGAATTCATGCGTCGTCGTCTCTAACG |
| XR4-NOTI-R | AAGGAAAAAAGCGGCCGCttaGTCGAAGAGCATGCGGAGGA |
| XR5-ECORI-F | CCGGAATTCATGGCGACCATCAAACTGAA |
| XR5-NOTI-R | AAGGAAAAAAGCGGCCGCttaAACGAACAGCGGGATTTTGT |
| XR6-ECORI-F | CCGGAATTCATGAAAGTTGACAACGCGAC |
| XR6-NOTI-R | AAGGAAAAAAGCGGCCGCttaAACCAGACGAGACTGCTGCA |
| XR7-ECORI-F | CCGGAATTCATGTCCCAGGTTTACGTTAC |
| XR7-NOTI-R | AAGGAAAAAAGCGGCCGCttaAACGAAGGTCGGGATTTCCG |
| XR8-ECORI-F | CCGGAATTCATGTCTACCGCGACCGCGTC |
| XR8-NOTI-R | AAGGAAAAAAGCGGCCGCttaAACGAAAACCGGGATGTTAG |
| XR9-ECORI-F | CCGGAATTCATGTCTACCACCCCAACCAT |
| XR9-NOTI-R | AAGGAAAAAAGCGGCCGCttaAACGAAGATCGGAATGTTGT |
| XR10-ECORI-F | CCGGAATTCATGTCTAGCATCGTTACGCT |
| XR10-NOTI-R | AAGGAAAAAAGCGGCCGCttaCGCGAAGATCGGCAGTTTCA |
| KMGAP-HINDIII-F | CCCAAGCTTGCATGCCCATTACCCGGAAT |
| KMGAP-R | TGTGATGTGTAAAAGTGTGT |
| KMGAP-CTXR-F | CACACACTTTTACACATCACAATGTCTACCACCCCAACCATCCCGACC |
| TER-HINDIII-R | CCCAAGCTTTCAATCAATGAATCGAAAATG |

**C**

**D**

**B**

**A**

**F**

**E**

**I**

**G**

**H**

**J**

**K**

Fig. S1 Plasmids schema used in this study.

**
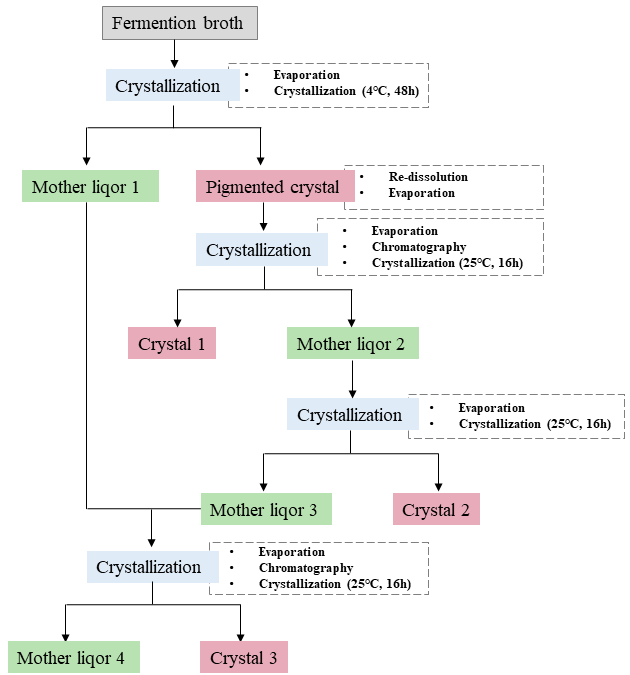
**

Fig. S2 Xylitol purification flow chart in this study.
